# High Mortality of Disseminated Non-Tuberculous Mycobacterial Infection in HIV-Infected Patients in the Antiretroviral Therapy Era

**DOI:** 10.1371/journal.pone.0151682

**Published:** 2016-03-17

**Authors:** Tetsuro Kobayashi, Takeshi Nishijima, Katsuji Teruya, Takahiro Aoki, Yoshimi Kikuchi, Shinichi Oka, Hiroyuki Gatanaga

**Affiliations:** 1 AIDS Clinical Center, National Center for Global Health and Medicine, Tokyo, Japan; 2 Center for AIDS Research, Kumamoto University, Kumamoto, Japan; Cambridge University, UNITED KINGDOM

## Abstract

**Background:**

Little information is available on the mortality and risk factors associated with death in disseminated non-tuberculous mycobacterial infection (dNTM) in HIV-infected patients in the ART-era.

**Methods:**

In a single-center study, HIV-infected dNTM with positive NTM culture from sterile sites between 2000 and 2013 were analysed. The clinical characteristics at commencement of anti-mycobacterial treatment (baseline) were compared between those who survived and died.

**Results:**

Twenty-four patients were analyzed. [The median CD4 27/μL (range 2–185)]. *Mycobacterium avium* and *M*. *intracellulare* accounted for 20 (83%) and 3 (13%) of isolated NTM. NTM bacteremia was diagnosed in 15 (63%) patients. Seven (29%) patients died, and NTM bacteremia was significantly associated with mortality (p = 0.022). The baseline CD4 count was significantly lower in the non-survivors than the survivors (median 7/μL versus 49, p = 0.034). Concomitant AIDS-defining diseases or malignancies were not associated with mortality. Immune-reconstitution syndrome (IRS) occurred to 19 (79%) patients (8 paradoxical and 11 unmasking), and prognosis tended to be better in unmasking-IRS than the other patients (n = 13) (p = 0.078). Patients with paradoxical-IRS had marginally lower CD4 count and higher frequency of bacteremia than those with unmasking-IRS (p = 0.051, and 0.059). Treatment with systemic corticosteroids was applied in 63% and 55% of patients with paradoxical and unmasking-IRS, respectively.

**Conclusion:**

dNTM in HIV-infected patients resulted in high mortality even in the ART-era. NTM bacteremia and low CD4 count were risk factors for death, whereas patients presented with unmasking-IRS had marginally better prognosis. IRS occurred in 79% of the patients, suggesting difficulty in the management of dNTM.

## Introduction

Non-tuberculous mycobacteria (NTM) have been recognized since early 1950s as a causative agent of various human diseases [1]. A substantial percentage of NTM infections are associated with immuno-deficient factors including human-immunodeficiency virus (HIV) infections, especially in patients with CD4 count of less than 50/μl [2]. In the pre-antiretroviral therapy (ART) era, it was estimated that up to 43% of AIDS patients had disseminated NTM infections, defined as isolation of NTM either in blood culture or mesenteric lymph nodes on autopsy [3]. The most common organism identified in this study was *Mycobacterium avium-intracellulare* complex (MAC), accounting for 86% of the disseminated NTM infections. However, following the introduction of both ART and primary prophylaxis, such as clarithromycin, the overall incidence of disseminated MAC infection in HIV-infected patients has dropped by 10-fold to about 2.5 cases per 1,000 person-years in the ART era [4, 5].

In the pre-ART era, the reported one-year mortality rate in HIV-infected patients with NTM bacteremia was a high as 71% [6]. The risk factors contributing to death in disseminated NTM infection included delay in antimycobacterial treatment and/or ART, and initial level of NTM bacteremia [7, 8]. To our knowledge, however, there is lack of information on the mortality and related risk factors in patients with disseminated NTM infection diagnosed in the ART-era, with one of the reasons being the aforementioned drop in the incidence of disseminated NTM infection [4, 5]. In this single-center study, we investigated the mortality and risk factors associated with death in disseminated NTM infection in HIV-infected patients diagnosed in the ART-era.

## Methods

The study subjects of this retrospective, single-center study were HIV-infected patients over 19 year-old who were diagnosed with disseminated NTM infection at AIDS Clinical Center, National Center for Global Health and Medicine, Tokyo, Japan, between January 1, 2000 and December 31, 2013. Our facility is one of the largest clinics for patients with HIV infection in Japan with more than 3,800 registered patients [9]. The diagnosis of disseminated NTM infection was based on positive culture of the mycobacterium from at least one sterile sites (e.g., blood, bone marrow, cerebrospinal fluid, synovial fluid, and/or lymph nodes) [10]. Patients with positive cultures of specimens from the respiratory tract (such as sputum), gastrointestinal tract (such as stool and gastric juice), or wound but negative cultures from sterile sites were excluded. Patients were also excluded if: 1) NTM was identified based on histopathology of sterile sites but with negative culture, 2) clinically or radiologically diagnosed patients without positive cultures who responded well to anti-mycobacterial drugs, 3) transferred to another facility or lost to follow-up within one year from initiation of anti-mycobacterial treatment, or 4) recurrent NTM infections which was first diagnosed before January 1, 2000.

The study was approved by the Human Research Ethics Committee of the National Center for Global Health and Medicine, Tokyo. The study was conducted according to the principles expressed in the Declaration of Helsinki. Informed consent was waived because this study only used the data gained from clinical practice. Patient records/information was de-identified prior to analysis.

### Measurements

The clinical characteristics of patients with disseminated NTM infection were collected from the medical records. They included age, sex, nationality, route of HIV transmission, baseline HIV status (CD4 cell count and HIV viral load) at the initiation of anti-mycobacterial treatment, sites of specimen from which NTM was cultured, concomitant NTM bacteremia, duration of anti-NTM treatment, concomitant immune-reconstitution syndrome (IRS), presence of other acquired immunodeficiency syndrome (AIDS)-defining diseases, such as cytomegalovirus end-organ diseases, and underlying chronic diseases, such as diabetes mellitus, malignancy, hepatitis C, and hepatitis B infection [10, 11]. Patients with IRS were further categorized into two sub-groups: those who presented with worsening of the disease being treated with anti-mycobacterial drugs after introduction of ART (paradoxical type) and those who presented with deterioration of previously-unrecognized infections after the introduction of ART (unmasking type) [12–14]. The clinical presentation was recorded at two points: at initiation of treatment for disseminated NTM infection (baseline) and at onset of IRS. The variables included fever (defined as maximum temperature 38.3°C or higher [15]), superficial lymphadenopathy, deep lymphadenopathy (defined as lymph nodes with diameter ≥2 cm on either computed tomography or sonography [16, 17]), cough, abdominal pain, and diarrhea.

The time intervals from the commencement of anti-NTM treatment to the commencement of ART and from the commencement of ART to diagnosis of IRS for each of the IRS-subgroups (if applicable) were determined in days. For patients treated with anti-mycobacterial drugs at the time of manuscript submission, the duration of anti-mycobacterial treatment was censored on June 1, 2015.

### Statistical analysis

The baseline characteristics at the start of anti-mycobacterial treatment were compared between the non-survivors and survivors using the Man-Whitney’s U-test or chi-square test (Fisher’s exact test when appropriate) for continuous or categorical variables, respectively. Furthermore, Man-Whitney’s U-test was used for comparison of live-or-death status according to the duration of anti-mycobacterial treatment, duration of ART, and time interval between start of anti-mycobacterial treatment and onset of IRS. Statistical significance was defined as two-sided *p* values <0.05. All statistical analyses were performed with The Statistical Package for Social Sciences ver. 21.0 (SPSS, Chicago, IL).

## Results

**Fig 1:**
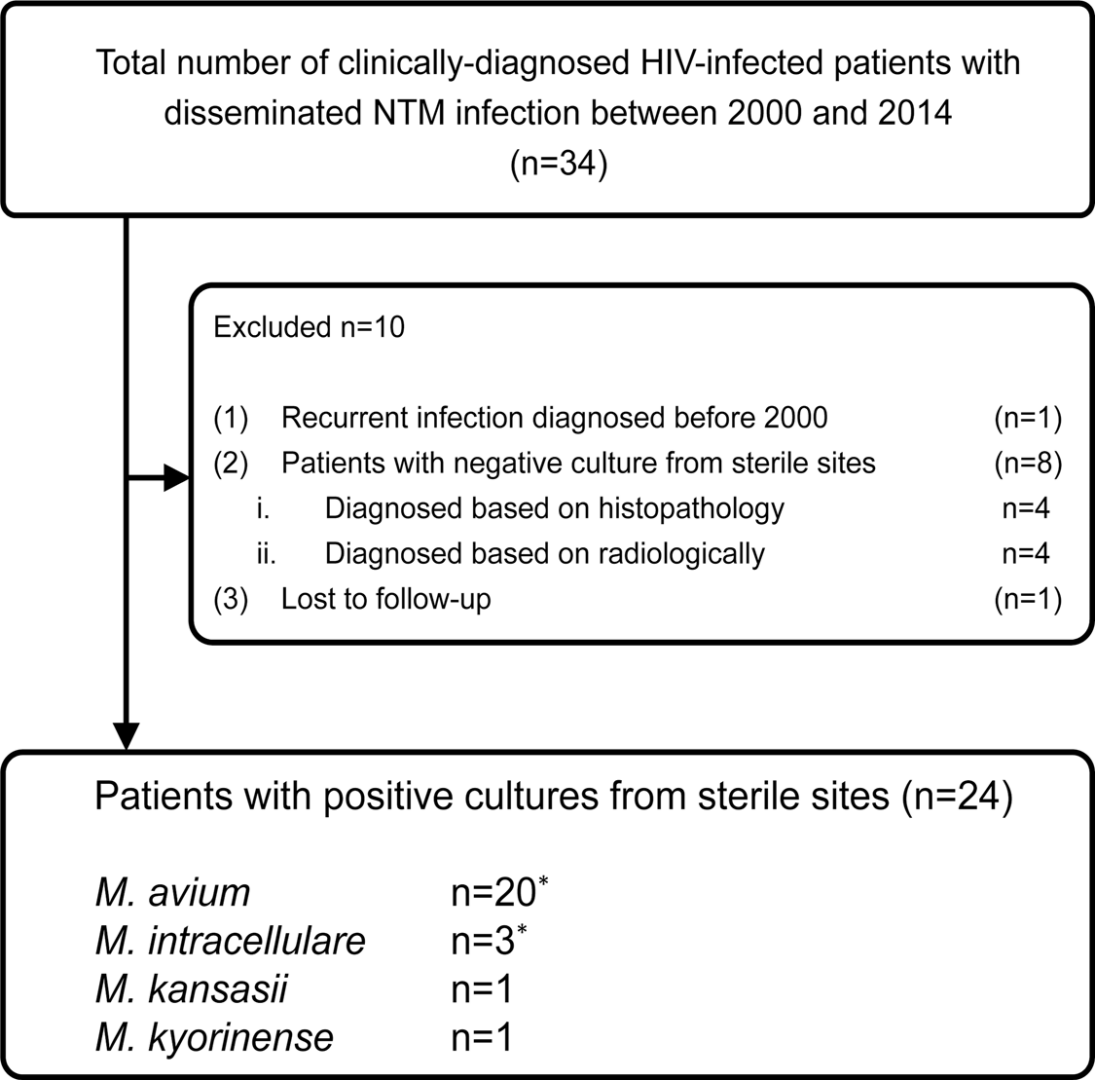
Patient enrollment. *Both *M*. *avium* and *M*. *intracellulare* were detected in blood culture of one patient.

Twenty-four patients were selected as the study patients (Fig 1). Twenty-two patients were males. 19 were infected with HIV through homosexual contact and 5 were infected with heterosexual contact. None of the 24 patients were injection drug users. All of the 24 were Japanese except one Myanmarese male and one Ugandan female (Table 1). The median age was 38 (range: 21–67), and the median CD4 count and HIV viral load at the time of initiation of anti-mycobacterial treatment were 27/μl (range: 2-185/μL), and 4.54 log_10_ copies/mL (range: 1.70–6.40 log_10_ copies/mL), respectively. Twelve patients were already on ART at initiation of anti-mycobacterial treatment, and 9 patients started anti-mycobacterial treatment before commencement of ART, while 3 patients died without receiving ART.

**Table 1:** Baseline characteristics of the study patients at initiation of anti-mycobacterial treatment.

Variables	n = 24
Age (years)^†^	38 (21–67)
Males, n (%)	22 (92%)
CD4 count (/μL)^†^	27 (2–185)
HIV-1 viral load (log_10_ copies/mL)^†^	4.54 (1.70–6.40)
Patients on antiretroviral therapy, n (%)	12 (50%)
NTM bacteremia, n (%)	15 (63%)
Initial anti-mycobacterial drugs	
Clarithromycin, rifabutin, and ethambutol	8 (33%)
Clarithromycin, rifabutin, ethambutol, and ciprofloxacin	4 (17%)
Clarithromycin, rifabutin, ethambutol, and levofloxacin	2 (8%)
Clarithromycin, rifampin, and ethambutol	2 (8%)
Clarithromycin and ethambutol	2 (8%)
Isoniazid, rifampin, and ethambutol	1 (4%)
Isoniazid, rifampin, ethambutol, and clarithromycin	1 (4%)
Clarithromycin, rifampin, ethambutol, and ciprofloxacin	1 (4%)
Clarithromycin, and ciprofloxacin	1 (4%)
Azithromycin, ethambutol, rifampin, and ciprofloxacin	1 (4%)
None	1 (4%)
Sites of specimens from which NTM was isolated	
Blood	15 (63%)
Lymph nodes	9 (38%)
Bone marrow	5 (21%)
Cerebrospinal fluid	2 (8%)
Synovial fluid	1 (4%)
Clinical signs and symptoms at diagnosis	
Fever	18 (75%)
Superficial Lymphadenopathy	18 (75%)
Deep Lymphadenopathy	15** (63%)
Cough	7* (29%)
Diarrhea	5* (21%)
Abdominal Pain	2* (8%)
Underlying diseases	
Malignancy	7 (29%)
Kaposi sarcoma	4 (17%)
Non-Hodgkin lymphoma	3 (13%)
Bowen disease	1 (4%)
Hepatitis B virus infection	4 (17%)
Hepatitis C virus infection	1 (4%)
AIDS-defining diseases (other than NTM infection)	
Cytomegalovirus end-organ diseases	20 (83%)
Pneumocystis pneumonia	15 (63%)
Kaposi sarcoma	12 (50%)
Non-Hodgkin lymphoma	4 (18%)
Candida esophagitis	3 (13%)
Toxoplasmosis	2 (8%)
Histoplasmosis	1 (4%)
Progressive multifocal leukoencephalopathy	1 (4%)

^†^Data are median (range).

*Data were not available for one patient.

**No records of computed tomography were available in two patients.

All 24 patients were tested for blood-culture, and 15 (63%) were confirmed to have bacteremia. The species of NTM isolated from sterile sites or blood were: *M*. *avium* in 20 patients (83%), *M*. *intracellulare* in 3 patients (13%), *Mycobacterium kansasii*, and *Mycobacterium kyorinense* each in 1 patient (4%). Both *M*. *avium* and *M*. *intracellulare* were detected on blood culture in one patient. The most common site, other than blood, from which NTM was isolated, was lymph nodes (38%), followed by bone marrow (21%), cerebrospinal fluid (8%), and synovial fluid (4%) (Table 1). The most common clinical symptom was fever (75%), followed by superficial lymphadenopathy (75%), deep lymphadenopathy (63%), cough (29%), diarrhea (21%), and abdominal pain (8%). The initial anti-mycobacterial regimens are listed in Table 1. Twenty-three patients received anti-mycobacterial drugs, and one patient died before initiation of anti-mycobacterial drugs.

Twenty (83%) patients had at least one AIDS-defining diseases other than NTM infection; cytomegalovirus end-organ disease in 15 (63%), pneumocystis pneumonia in 12 (50%), Kaposi sarcoma in 4 (13%), non-Hodgkin lymphoma in 3 (13%), candida esophagitis in 2 (8%), toxoplasmosis in 1 (4%), histoplasmosis in 1 (4%), and progressive multifocal leukoencephalopathy in 1 (4%). Twelve patients (50%) had at least one underlying condition; malignancy in 7 (29%), hepatitis B virus infection in 4 (17%), diabetes mellitus in 2 (8%), and hepatitis C virus infection in 1 (4%).

The clinical characteristics of the seven patients who died and the 17 survivors are compared in Table 2. Three (13%) patients died before the introduction of ART. The median time from diagnosis of disseminated NTM infection to death for the 7 non-survivors was 224 days (range 0 to 777). NTM bacteremia was diagnosed in all 7 (100%) patients of the death group compared with only 8 (47%) of the latter group, indicating that NTM bacteremia was significantly associated with death (p = 0.022). The baseline CD4 count was significantly lower in the non-survivors than in the survivors [median 7/μL (range 4–68) versus 49 (2–185), p = 0.034], although HIV viral load was not different between the two groups (p = 0.418). Time to blood culture positivity was not different between the two groups (p = 0.318). The presence of at least one concomitant AIDS-defining disease other than NTM infection was not associated with death (p = 0.283). Neither cytomegalovirus end-organ diseases nor pneumocystis pneumonia nor underlying malignancy were associated with death (p = 0.669, p = 1.000, and p = 0.134, respectively). Of the 7 patients who died, 3 died of disseminated NTM infections. Four had concomitant non-Hodgkin lymphoma and 1 had histoplasmosis; these comorbidities could also have contributed to their deaths.

**Table 2:** Characteristics of non-survived and survived patients.

Characteristics	Non-survived (n = 7)	Survived (n = 17)	p-value
Age (years)^†^	36 (32–67)	38 (21–64)	0.757
NTM bacteremia, n (%)	7 (100%)	8 (47%)	0.022
Time to blood culture positivity (days)^†^	13 (5–25)	10 (3–19)	0.318
CD4 count at commencement of anti-mycobacterial treatment (/μL)^†^	7 (4–68)	49 (2–185)	0.034
HIV-1 viral load at commencement of anti-mycobacterial treatment (log_10_ copies/mL)^†^	5.15 (1.70–5.88)	3.69 (1.70–6.40)	0.418
Underlying malignancy, n (%)	4 (57%)	3 (18%)	0.134
Concomitant AIDS-defining disease (other than NTM infection), n (%)	7 (100%)	13 (76%)	0.283
Cytomegalovirus end-organ disease	5	10	0.669
Pneumocystis pneumonia	3	9	1.000
Patients with IRS, n (%)	3 (43%)	16 (94%)	
Paradoxical IRS	2	6	
Unmasking IRS	1	10	
Patients without IRS, n (%)	4 (57%)	1 (6%)	
Patients who received ART	1	1	
Patients who did not receive ART	3	0	
Time interval between commencement of ART and onset of IRS^†^	59 (7–63) (n = 3)	17 (6–270) (n = 16)	0.385
Duration of anti-mycobacterial treatment (days)^†^	224 (0–777)	679 (128–1875)	

^†^Data are median (range).

IRS: immune-reconstitution syndrome, ART: antiretroviral therapy.

IRS occurred in 19 (79%) patients; 8 with paradoxical IRS and 11 with unmasking IRS. Table 3 provides a comparison of the clinical characteristics of the unmasking IRS group, paradoxical IRS group, and non-unmasking IRS group including all study patients other than those presented with unmasking IRS. In patients with paradoxical IRS, the median time interval between the start of anti-mycobacterial drugs and ART was 52 days (range; 4–147). The interval between the start of ART and onset of IRS was 16 days (range: 7–59) for the paradoxical group and 21 days (range: 6–270) for the unmasking group, and was not different between the two groups (p = 0.310). The median CD4 count was marginally higher in the unmasking group than in the paradoxical group [72/μl (range 10–185) versus 7.5/μl (range 2–164), p = 0.051], and was significantly higher in the unmasking group than in the non-unmasking IRS group [72/μl (range 10–185) versus 7/μl (range 3–164), p = 0.014]. Bacteremia occurred more frequently in the non-unmasking IRS group than in the unmasking group [11 of 13 (85%) in the non-unmasking group and 4 of 11 (36%) in the unmasking group, p = 0.033], and occurred marginally more frequently in the paradoxical IRS group than in the unmasking IRS group [7 of 8 (88%) in the paradoxical group and 4 of 11 (36%) in the unmasking group, p = 0.059]. There was no patient in the unmasking IRS group that had received primary prophylaxis before the start of anti-mycobacterial treatment. Eight out of 11 in the unmasking IRS group had been tested for blood culture before the diagnosis of disseminated NTM infection and none was positive. Abdominal pain and diarrhea were more frequently observed in the paradoxical group than in the unmasking group (p = 0.002 and 0.047). Five (63%) of 8 patients with paradoxical IRS required systemic corticosteroids for the management of IRS, whereas 6 (55%) of 11 patients with unmasking IRS required such therapy. While there was no significant difference in the mortality rate between the two IRS subgroups (p = 0.546), the unmasking IRS group had marginally lower mortality than in the non-unmasking IRS group [1 of 11 (9%) in the unmasking group and 6 of 13 (46%) in the non-unmasking group, p = 0.078].

**Table 3:** Comparative characteristics of patients presented with unmasking and paradoxical immune-reconstitution syndrome, and patients other than those who presented with unmasking immune-reconstitution syndrome.

Characteristics	Unmasking IRS group (n = 11)	Paradoxical IRS group (n = 8)	Non-unmasking group (n = 13) ^††^	p value (Unmasking vs. Paradoxical)	p value (Unmasking vs. Non-unmasking^††^)
Age (years)^†^	38 (21–64)	37 (25–58)	37 (25–67)	0.840	0.642
CD4 count (/μL) at initiation of anti-mycobacterial treatment^†^	72 (10–185)	7.5 (2–164)	7 (3–164)	0.051	0.014*
HIV viral load (log_10_ copies/mL) at initiation of anti-mycobacterial treatment^†^	1.70 (1.70–4.97)	5.70 (4.32–6.40)	5.15 (2.08–6.40)	<0.001*	<0.001*
NTM bacteremia, n (%)	4 (36%)	7 (88%)	11 (85%)	0.059	0.033*
Time interval from initiation of ART and onset of IRS (days)^†^	21 (6–270)	16 (7–59)	n/a	0.310	n/a
Presentation at onset of IRS					
Fever, n (%)	7 (63%)	6 (75%)	n/a	1.000	n/a
Deterioration of superficial lymphadenopathy, n (%)	7 (63%)	5 (63%)	n/a	1.000	n/a
Deterioration of deep lymphadenopathy, n (%)	6 (55%)	2** (25%)	n/a	0.620	n/a
Cough, n (%)	3 (27%)	0* (0%)	n/a	0.228	n/a
Abdominal pain, n (%)	0 (0%)	5* (63%)	n/a	0.002*	n/a
Diarrhea, n (%)	1 (9%)	4* (50%)	n/a	0.047*	n/a
Systemic corticosteroids, n (%)	6 (55%)	5 (63%)	n/a	1.000	n/a
Death	1 (9%)	2 (25%)	6 (46%)	0.546	0.078

^†^Data are median (range).

^††^The non-unmasking group consists of the 8 paradoxical IRS patients and 5 without any IRS.

IRS: immune-reconstitution syndrome, ART: antiretroviral therapy.

*Data were not available for one patient.

** No records of computed tomography were available in two patients.

## Discussion

We evaluated the mortality and risk factors for death in HIV-infected patients with disseminated NTM infection diagnosed between 2000 and 2013 at a large HIV referral center, and showed a high overall mortality (29%; 7 out of 24 patients), even in the ART era. Death was significantly associated with NTM bacteremia (p = 0.022) and those patients who died had lower CD4 count at the start of anti-mycobacterial treatment than those who survived (p = 0.034). On the other hand, patients who presented with the unmasking form of IRS appeared to have better prognosis than the other study patients (p = 0.078). NTM-IRS was diagnosed in 79% of the patients and of these, 58% required systemic corticosteroids therapy, suggesting difficulty in the management of NTM infection. Notably, unmasking IRS was diagnosed in 46% of the study patients, which also suggested that the diagnosis of disseminated NTM infection can be difficult before the introduction of ART. Although the incidence of disseminated NTM infection among HIV-infected patients has substantially decreased after the introduction of ART and use of primary prophylaxis, the results of the present study showed that disseminated NTM infection is a severe, life-threatening opportunistic infection; such infection is associated with high mortality and other concurrent AIDS-defining illnesses (in >80% of the patients), and requires long-term treatment [among the survivors, anti-NTM treatment was prescribed for median of 679 days (range 128–1875)].

To our knowledge, this is the first study that focused on the mortality and associated factors in disseminated NTM infection with HIV infection in the ART era. Most reports on HIV-infected disseminated NTM infection were from the pre-ART era or included a high percentage of patients from such era [6, 7, 12, 18]. Due to the low incidence of disseminated NTM infection in the ART era, only few studies have been published on this disease [19, 20]. However, one study from Taiwan, which examined mortality in 40 disseminated NTM patients (22 with HIV infection and 18 without HIV infection), included patients with positive cultures from non-sterile sites, such as respiratory tract, that could result in the inclusion of patients with false diagnosis of disseminated NTM infection, and importantly, that study did not describe the mortality among HIV-infected patients [19]. Another study from the US, which investigated the prevalence of mycobacteria disease among hospitalized HIV-infected patients, also included clinically-diagnosed patients with disseminated NTM infection, and also was conducted during 2001 to 2002 [20], the time the ART was just developed and was not effective and tolerable as today. Thus, another strength of the present study is that we only included patients with culture-positive NTM infection from a sterile site, such as blood, bone marrow, and lymph nodes, and successfully excluded patients with clinical or probable diagnosis of disseminated NTM infection.

The present study identified NTM bacteremia to be a risk factor for mortality in disseminated NTM infection, which seems consistent with the finding from pre-ART era; that the initial level of NTM bacteremia, measured by the number of colony forming unit, is a risk factor for mortality among HIV-infected patients with NTM bacteremia [7]. It is of interest that, in the present study, the time to culture positivity, which could also be a marker of the amount of NTM in the sterile site, was not associated with mortality (p = 0.318). Unfortunately, it is difficult to interpret this discrepancy with a small number of the study patients (n = 24) and with the present study that included not only patients with NTM bacteremia, but also those with positive cultures from other sterile sites, such as bone marrow, based on the well-established criteria for definite diagnosis of disseminated NTM infection [10]. However, these findings might suggest that NTM bacteremia, rather than culture positivity from other sterile sites, reflect the severity of the disease.

Our study also showed that CD4 count of the non-survivors at the commencement of anti-mycobacterial treatment was lower than that of survived patients. Although low CD4 count, particularly CD4 count less than 50/μl, is a well-known risk factor for the development of disseminated NTM infection [2], to our knowledge, there are no studies that showed such relationship between CD4 count and mortality in HIV-infected disseminated NTM infections.

IRS occurred to almost 80% of the patients, and the majority of patients with IRS required systemic corticosteroid therapy for the management of IRS. These findings suggest the difficulty in managing disseminated NTM infections in HIV-infected patients. Also, 11 of the 19 IRS patients (46% of the total patients) presented with unmasking IRS, showing that almost half of the disseminated NTM infections were unrecognized at the time of initiation of ART. Disseminated NTM infection should always be one of the differential diagnosis for patients with fever or lymphadenopathy (both of which were observed in more than 50% of patients with unmasking IRS) after initiation of ART, especially in those with CD4 count less than 200/μl.

Apart from strengths described above, we need to acknowledge a limitation. The number of patients was small due to the low incidence of disseminated NTM infection in the ART era and conduct of the study in a single center. However, our study included only patients with positive cultures from the sterile sites and excluded those clinically-diagnosed based on cultures from non-sterile sites, such as respiratory tract, radiology, or pathology. This is important considering that the clinical symptoms and signs of disseminated NTM infection is often non-specific; such as fever, lymphadenopathy, and diarrhea, and a definite diagnosis of the disease is difficult, since the study showed that 46% of the patients were diagnosed only after the introduction of ART, when they presented with an unmasking IRS.

In conclusion, the present study showed that disseminated NTM infection in HIV-infected patients is associated with high mortality even in the ART era. NTM bacteremia and low CD4 count were significantly associated with mortality, and patients who presented with disseminated NTM infection as unmasking IRS appeared to have better prognosis. Almost 80% of the patients experienced IRS, suggesting difficulty in the management of such infection.

## Supporting Information

S1 Dataset Dataset of the study patients. (XLSX)
